# The survival processing advantage in memory using virtual reality versus traditional desktop display: Does it make a difference?

**DOI:** 10.3758/s13421-025-01846-2

**Published:** 2026-02-20

**Authors:** Patrick Bonin, Alain Méot, Stéphane Argon, Jean-Michel Boucheix, Gaëtan Thiebaut

**Affiliations:** 1 Université Bourgogne Europe, CNRS, LEAD UMR5022, Pôle AAFE–Esplanade Erasme, 21065 Dijon Cedex, France; 2 LAPSCO-CNRS, UMR6024, Université Clermont-Auvergne, Clermont-Ferrand, France; 3 Laboratoire de Psychologie (UR3188), Université Marie & Louis Pasteur, Besançon, France

**Keywords:** Adaptive memory, Survival processing, Virtual reality, Elaboration

## Abstract

The survival processing advantage refers to the fact that words processed in relation to survival (e.g., finding food) are memorized better than words processed in a context that is not evocative of survival (e.g., moving to a foreign country). This effect has been extensively studied in laboratory tasks in which participants have to imagine a survival situation rather than being placed in a “supporting perceptual context.” In the present study, we aimed to investigate the survival effect using virtual reality and compare it with a traditional desktop display. Using virtual reality technology, we replicated the survival processing advantage found in Wang et al.’s (Survival processing advantage demonstrated with virtual reality-based survival environment: A promising tool for survival processing research. *Memory & Cognition, 51*[1], 129–142, 2023) study. More importantly, the survival effect obtained with virtual reality was no greater than that obtained using more conventional means (i.e., a desktop display). The findings are discussed in relation to the issue of the optimality of the survival effect and the way proximate mechanisms are involved.

**Supplementary Information:**

The online version contains supplementary material available at 10.3758/s13421-025-01846-2.

## Résumé en français

L’avantage du traitement survie désigne le phénomène selon lequel les mots traités dans un contexte lié à la survie (e.g., recherche de nourriture) sont mieux mémorisés que ceux traités dans un contexte non évocateur de survie (e.g., déménagement dans un pays étranger). Cet effet a été largement étudié au moyen de tâches expérimentales conduites en laboratoire, dans lesquelles les participants doivent se représenter mentalement une situation de survie, plutôt que d’être placés dans un contexte perceptif soutenant directement le traitement de l’information. La présente étude visait à examiner l’effet survie en mémoire à l’aide de la réalité virtuelle et à le comparer à une présentation traditionnelle sur écran d’ordinateur. Grâce à l’utilisation de la réalité virtuelle, nous avons répliqué l’avantage du traitement survie rapporté par Wang et al. (2023). Toutefois, l’effet survie observé en réalité virtuelle ne s’est pas avéré supérieur à celui obtenu avec des moyens plus conventionnels (i.e., présentation sur écran d’ordinateur). Ces résultats sont discutés en lien avec la question de l’optimalité de l’effet de survie et du rôle des mécanismes proximaux sous-jacents.

Words processed in relation to survival problems are remembered better than words processed in relation to nonsurvival problems, such as moving to a foreign country. This memory effect, known as the survival processing advantage or survival effect, is now a well-established finding in the literature. It has been obtained virtually in laboratory experiments involving desktop displays but has been obtained using virtual reality in only a single study—namely, that conducted by Wang et al. (2023). The present research focuses precisely on the survival effect in relation to virtual reality. This memory phenomenon was discovered relatively recently (Nairne et al., 2007) by researchers who adopted an evolutionary perspective on memory. According to James Nairne, memory was shaped by natural selection, enabling our hunter–gatherer ancestors to solve survival-related problems, such as remembering where to find food and drinking water, or where dangerous animals roam. In other words, memory is an adaptation (Nairne, 2016, 2022). Since the pioneering studies by Nairne et al. (2007, 2008; Nairne & Pandeirada, 2010), the survival effect has been the subject of extensive research, attesting to its robustness and providing a clearer picture of its characteristics (see Kazanas & Altarriba, 2015; Nairne & Pandeirada, 2016; for reviews, Scofield et al., 2018, for a meta-analysis).

In the classic experimental task used to demonstrate the survival effect, adults are asked to use Likert scales to rate the relevance of words in relation to an imaginary survival scenario (e.g., being stranded in the grasslands of a foreign land without any basic survival materials). The results of a subsequent memory test for the rated words are then compared either with deep encoding tasks, such as pleasantness ratings, self-reference or imagery (Nairne & Pandeirada, 2008), or with “control” scenarios that do not involve survival, such as finding a new home when moving to a foreign country (e.g., Nairne et al., 2007). Another important aspect of the survival effect is that it occurs in different contexts. For example, it has been observed in ancestral contexts such as the African savannah (e.g., Nairne et al., 2007; Nairne & Pandeirada, 2010; Weinstein et al., 2008), as well as in modern survival contexts such as the city (Soderstrom & McCabe, 2011). Indeed, the survival effect has been demonstrated in a variety of imagined locations, including the desert, the jungle, the sea, space (Kostic et al., 2012), and even in response to fictious threats such as zombies and ghosts (Bonin et al., 2019). As mentioned above, one important feature that virtually all studies on the survival effect have in common is that they use *imagination* and *desktop displays*.

## Virtual reality and memory

Virtual reality (VR) is an innovative technology that can be used in psychological research to increase ecological validity beyond levels normally achievable in laboratory research today (see Kothgassner & Felnhofer, 2020, for a discussion of the relation between ecological validity and virtual environments; Holleman et al., 2020, for a critical review of the concept of ecological validity in psychology). In particular, VR has been claimed to be “a powerful means to enhance the ecological validity of memory research by providing realistic virtual environments” (p. 1) (Reggente et al., 2018). VR can be particularly useful for investigating certain aspects of cognitive functioning (Ventura et al., 2019), as laboratory procedures can be highly constrained and fail to reflect the multidimensional nature and complexity of real-life experiences. To date, few studies have considered using VR to explore cognitive mechanisms linked to survival. For example, a study by Yuan et al. (2018) showed that threatening stimuli (e.g., spiders, snakes) were detected faster than nonthreatening stimuli (e.g., flowers, squirrels) in a virtual grove environment. VR can also be used to study memory, especially episodic memory (e.g., Cadet & Chainay, 2020; Dinh et al., 1999; Plancher et al., 2013; Schöne et al., 2019; Smith, 2019; Smith & Mulligan, 2021) as we shall describe later in the Introduction.

When considering how VR can be used to study behavior, it is important to take various parameters into account: immersion, presence, and realism. Jung and Lindeman (2021) define realism as the extent to which the virtual environment emulates the real world. In contrast, “presence” (or “illusion,” as they consider it to be an equivalent term) is defined as the overall subjective quality of the VR experience. Dinh et al. (1999) investigated the impact of multisensory VR experiences using a large number of sensory modalities, such as tactile, olfactory, audio, and visual cues. They found that increasing the number of sensory input modalities in a virtual environment enhances the sense of presence. Immersion can be objectively defined and bears out the idea that “more is better”: Wide, high-resolution field-of-view HMDs and wide, accurate, six-degrees-of-freedom tracking provide more immersion than screen-based VR, and the same is true of spatial audio cues when compared with binaural or monaural audio cues (Jung & Lindeman, 2021). According to Smith (2019; see also Smith & Mulligan, 2021), more immersive VR systems may promote better episodic memory performance in certain instances. Indeed, some studies have shown that VR systems which promote immersion also increase memory performance (e.g., Dinh et al., 1999; Krokos et al., 2019; Schöne et al., 2019; however, see Cadet & Chainay, 2020; Gamberini, 2000). Interactions with the environment are another important component of immersiveness. These can range from having full control over navigation and the ability to manipulate objects to simply being able to turn one’s head to observe different aspects of the virtual environment (Smith, 2019). Increased VR interactivity has been found to play a role in memory performance such as free recall or recognition (e.g., Sauzéon et al., 2012).

Turning to the concept of “presence,” which is defined as the subjective feeling of being actually transported into the virtual environment (Smith, 2019), it is generally assumed that more immersive VR technologies produce a stronger sense of presence (Cadet & Chainay, 2020; Makransky et al., 2019; North & North, 2016). While these two concepts are closely related, it is possible to distinguish between them. According to Smith (2019), immersion is determined by the objective characteristics of the VR system, such as the amount of visual detail it allows. In contrast, presence refers to the subjective aspect of immersion and translates into a spectrum of “transport” in the virtual universe. The findings regarding the relationship between memory and presence are mixed. Lin et al. (2002) found increased immersion resulting from a greater field-of-view to be significantly related to increased presence and memory, whereas other authors found either no positive correlation between presence and memory performance (Mania & Chalmers, 2001), a negative relationship (Makransky et al., 2019), or no mediating effect of presence, suggesting a degree of independence between immersion, presence, and their impact on memory performance (Smith & Mulligan, 2021). Studies testing memory in VR have thus far failed to demonstrate a stable link between “presence” and “memory performance” (e.g., Ahn et al., 2022; Smith & Mulligan, 2021). Regarding the proximate memory mechanisms underlying the impact of immersion and presence, respectively, it has been suggested that immersion may operate via item processing (e.g., Sauzéon et al., 2012), while presence would lead to a greater level of attention being devoted to the virtual environment (Smith & Mulligan, 2021). According to Schöne et al. (2019), memory traces constructed during an immersive sensation of a virtual experience are characterized by richer content and more elaborate associative networks. Related to Schöne et al.’s (2019) assumption is the hypothesis that survival processing also promotes elaboration to a much greater extent than other types of “control condition,” such as “moving” or “pleasantness.” Elaboration is a proximate mechanism often put forward to explain the survival processing advantage (e.g., Howe & Derbish, 2014; Kroneisen & Erdfelder, 2011; Otgaar et al., 2015). The idea is that items processed in connection with survival-related issues lead to the creation of more numerous and richer memory traces than those generated in the control conditions (Bell et al., 2015; Kroneisen et al., 2013, 2024; Röer et al., 2013). In support of this hypothesis, Röer et al. (2013) found that survival processing led to the generation of more ideas than nonsurvival processing, for example moving to a foreign country.

## Objectives of the present study

The study by Wang et al. (2023), which first identified the survival effect in a virtual reality context, and more specifically in virtual ancestral and modern survival contexts, forms the basis for the present research. In this seminal study, adults were asked to rate the relevance of different words while immersed in a virtual reality environment corresponding to either an ancestral survival scenario (e.g., grasslands), a modern survival scenario (e.g., battlefield), or a domestic virtual space representing a house-moving situation. The results showed that the survival effect can emerge from “perceptual experiences” instead of just from “imagination-based” methodology.

Our first aim was to replicate Wang et al.’s (2023) results concerning the survival effect in VR, as well as to study it under more optimal conditions using one of the latest generation immersive technologies (Meta Quest 2; see the Procedure subsection of Study 2 for details). Meta Quest 2 is a significant improvement on the Oculus Rift DK2 used in Wang et al.’s (2023) study, particularly in terms of the quality of sensory information. Certain studies have shown that more immersive VR systems promote better episodic memory performance (Smith, 2019; Smith & Mulligan, 2021). Therefore, it is reasonable to assume that the latest versions of Unity3d and Meta Quest 2, which offer greater immersion, provide greater encoding richness. However, we lack data directly comparing these two devices.^1^ The device offered two distinct virtual environments: A natural environment that simulated potentially dangerous outdoor elements and a domestic environment that recreated a house-moving context without threatening elements. These virtual environments were designed to maximize sensory experience and participant engagement. In our study, they included multimodal information to enhance immersion, such as noises characteristic of a natural environment (e.g., wind and crickets) or a domestic context (e.g., outdoor road traffic), as well as its subjective counterpart, presence. By significantly enriching the sensory quality of the tested virtual environments (survival vs. moving), and thereby increasing the intensity of the presence experienced by participants, the survival effect would emerge in conditions as similar as possible to those encountered by our ancestors in the distant past.

**Fig. 1:**
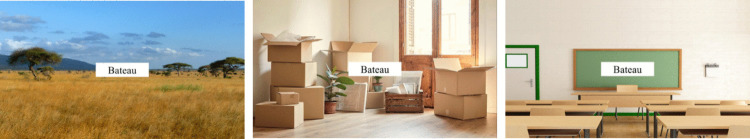
Illustration of the three encoding conditions used in Study 1 (from left to right: ancestral, moving, and school/neutral context). The French word “bateau” means “boat”

The second aim was to compare the survival effect in VR to that obtained in more conventional laboratory conditions, as this was not done in Wang et al.’s (2023) study. We therefore designed an initial benchmark study (Study 1) using a more traditional laboratory approach with a desktop display. However, we modified the standard procedure somewhat by adding a visual context to the presentation of the words in the different experimental conditions so that we could compare this approach more directly with VR. As illustrated in Fig. 1, an image of the African savannah context appeared on a computer screen in the survival condition, while the image in the moving condition showed a room with storage boxes stacked inside it. The context used for the pleasantness condition was a neutral classroom with chairs and a chalkboard, on which words were presented. Study 1 thus made it possible to test the survival effect under more conventional conditions, in which presence is assumed to be lower.

One possible hypothesis is that the survival effect will be greater than that obtained in more conventional laboratory conditions because a sensorially rich context favors more distinctive encoding (i.e., more item-specific processing; Einstein & Hunt, 1980), which is more beneficial for subsequent memory retrieval than a classic computer screen situation, as this context is less “rich” in stimulation. This hypothesis is consistent with the “richness of encoding” account often used to explain the memory survival effect (Bell et al., 2015; Kroneisen & Erdfelder, 2011; Kroneisen et al., 2013, 2024; Röer et al., 2013). As described above, this hypothesis holds that the experimental context of the survival scenario stimulates the generation of more numerous and distinctive memory traces than the control contexts generally used, such as a moving scenario. As VR permits the creation of more detailed graphical environments and sensorially and cognitively richer interactions, we hypothesized that this augmented realism might reinforce the adaptive bias achieved by the survival effect in memory. In other words, using VR in a survival context should permit richer encoding and thus produce better memory retention than more conventional encoding conditions, such as static 2D images on a computer screen and a silent sound environment in a cubicle. More specifically, the survival processing advantage measured by correct free recall should be greater when using VR than with a traditional computer-screen context. Assuming that more elaboration takes place in VR than with a traditional desktop display, we can also expect more clustering in VR than on a computer screen, particularly in the survival encoding condition. This, however, presupposes that elaboration includes relational processing (e.g., Einstein & Hunt, 1980; Howe & Derbish, 2014) and that elaboration does not primarily refer to the distinctiveness of the processing. According to Smith and Mulligan (2021), presence leads to a greater level of attention being devoted to the virtual environment. Thus, an equally possible hypothesis is that the richness-of-encoding boost in a VR environment applies similarly to all encoding conditions, not just the survival encoding condition. It is therefore possible to predict an overall increase in memory performance in VR encoding conditions compared with the more conventional screen-based encoding conditions.

## Study 1: Survival effect in a “classic” experimental setting (presentation on a computer screen in a silent cubicle)

### Method

#### Participants

A total of 120 participants (105 women, *M* = 19.91 years, *SD* = 2.16) from Université Bourgogne Europe in Dijon took part in the study, after seven participants had been excluded from the initial pool of 127 participant: Two were taking neuroleptics, one was not a native speaker of French, one had a very low recall rate, and three had significantly longer reaction times than the other participants. They were randomly assigned to one of three groups (*n* = 40 in each group) which experienced three different encoding conditions (survival vs. moving vs. pleasantness). All participants were native speakers of French and none were taking medication known to affect the central nervous system. Participants received course credits for their participation. All the study procedures were approved by the Statutory Ethics Committee of the University Bourgogne Franche-Comté.

The number of participants was chosen on the basis of Scofield et al.’s (2018) meta-analysis of the survival processing advantage in memory. The set of studies using between-subjects designs included in their analysis had a mean of 37.5 participants per group. Given an ${n}_{p}^{2}$ = .075—a value situated at the center of the survival ${n}_{p}^{2}$ effect size interval—estimated between .06 and .09—the sample size required to obtain a power of .8 in a unilateral test at the .05 level was equal to 120 (3 × 40).^2^

#### Stimuli

The word list was taken from Bonin et al.’s (2025a) Study 1, in which words were selected to be moderately relevant to various survival issues, such as finding food and water (*M* = 2.9, *SD* = .43), protecting from predators (*M* = 2.9, *SD* = .50), and avoiding pathogens (*M* = 2.18, *SD* = .42). Thirty words belonging to four semantic categories were used: 13 were objects (e.g., rake, lasso), eight were animals (e.g., bear, snake), five were means of transport (e.g., boat, tractor), and four were shelters (e.g., cave, castle). A list of the words, their English translations, and their semantic categories can be found in Bonin et al. (2025a).

To make it possible to compare the results of Study 1 with those obtained using VR, the words were presented alongside visual prompts corresponding to the encoding condition. The survival scenario showed a photograph of an African savannah with tall grass and acacia trees. The moving scenario displayed a photograph of a room filled with boxes. The photograph used for the pleasantness scenario was chosen for its neutrality; it depicted a classroom with tables and a board (see Fig. 1 for illustrations of the different photographs).

#### Apparatus

The experiment script was created using PsyScope (Cohen et al., 1993), which was run on an Apple computer (iMac 20-in. screen, 2.66 GHz Intel Core 2 Duo processor) with a resolution of 1,680 × 1,050.

#### Procedure

Participants were tested individually in a quiet room. Written informed consent was obtained from all the participants prior to the start of the experiment. First, we collected demographic information, including age, gender, native language, educational level, and neuroleptic use. Participants were then randomly assigned to one of the three encoding conditions: “survival,” “moving,” or “pleasantness.” The participants were then given the instructions for the rating task, which was presented on a computer screen.

The scenario for the survival rating task was adapted from those used in previous experiments (Bonin et al., 2024; Nairne et al., 2007). Participants were asked to imagine being stranded in the grasslands of a foreign land without any basic survival materials. They were told that, over the next few months, they would need to find steady supplies of food and water, and protect themselves against predators. In the moving scenario, participants were asked to imagine preparing to move abroad. Over the next months, they would need to find and purchase a new home and transport their belongings. In both scenarios, participants had to rate words for their relevance to the corresponding situation using Likert scales (1 = the word is “totally irrelevant” to 5 = “extremely relevant”), which they did by pressing a corresponding number on the keyboard. In the pleasantness condition, participants had to rate the words for their pleasantness (1 = “very unpleasant” to 5 = “very pleasant”). The words were randomly presented one-by-one in the center of the photograph corresponding to the encoding context (Fig. 1). They remained on display until the participant responded (there were no practice trials). Participants were instructed to rate the words spontaneously and informed that there were no right or wrong answers. Following the encoding task, the participants undertook two distractor tasks that lasted about three minutes: the “plus–minus” task from Jersild (1927) and Spector and Biederman (1976), and the “X–O” letter-comparison task (Salthouse et al., 1997). The surprise recall test took place immediately afterwards. The participants were given five minutes to write down the words that had been presented to them earlier, in any order they liked. They were then asked to respond to the question “How real did the situation you were in feel to you?” using a Likert scale ranging from 1 (“not at all real”) to 7 (“very real”). They were then debriefed regarding the research goals. The entire experimental session lasted approximately 20 minutes.

#### Data analyses

Percentages of correct recall, numbers of intrusions, ARC clustering scores, encoding times, relevance ratings and ‘feeling real’ scores were considered in turn as a dependent variable in a by-participants analysis of variance using the scenarios’ conditions as an independent variable, complemented by paired comparisons between conditions. Encoding times, relevance ratings and feeling real scores were also included one by one as covariates in order to check if the mnemonic pattern was the same when these factors were controlled for.

### Results

#### Correct recall

The effect of condition was significant, *F*(2, 117) = 10.2, *p* < .0001, ${n}_{p}^{2}$ = .149. No reliable difference was observed between the moving scenario (*M* = .45, *SD* = 0.10) and the pleasantness condition (*M* = .42, *SD* = 0.14), *t*(117) = 1.22, *p* = .2266, *d* = 0.272. More words were recalled when words were rated in the survival scenario (*M* = .54, *SD* = 0.13) than in the moving scenario, *t*(117) = 3.16, *p* = .002, *d* = 0.707, or the pleasantness condition, *t*(117) = 4.37, *p* < .0001, *d* = 0.979. The number of extralist intrusions (*M* = 0.65, *SD* = 1.03) was low and did not differ significantly across conditions (survival: *M* = 0.68, *SD* = 1.07; moving: *M* = 0.80, *SD* = 1.20; pleasantness: *M* = 0.48, *SD* = 0.78), *F*(2, 117) = 1, *p* = .3692, ${n}_{p}^{2}$ = .017.

#### Clustering

We calculated category clustering using the adjusted ratio of clustering, or ARC score (Roenker et al., 1971). This score reflects the extent to which items from the same semantic category are recalled together. An ARC score of 1 indicates perfect clustering, while a score of 0 indicates clustering at chance level. ARC scores were above 0 in all three conditions—survival: *t*(39) = 3.86, *p* = .0004, *d* = 0.61, *M* = .18, *SD* = 0.29; moving: *t*(39) = 2.79, *p* = .0082, *d* = 0.44, *M* = .12, *SD* = 0.28; pleasantness: *t*(39) = 2.71, *p* = .01, *d* = 0.428, *M* = .16, *SD* = 0.38—and there were no reliable differences between them, *F*(2, 117) = 0.28, *p* = .7518, ${n}_{p}^{2}$ = .005. The inclusion of the semantic category classification of intrusions in the calculation of the ARC scores did not alter the pattern of results within or between conditions.

#### Encoding times

There was a significant difference in the time taken to rate the words between the three encoding conditions, *F*(2, 117) = 4.54, *p* = .0126, ${n}_{p}^{2}$ = .072. Rating times were lower in the moving condition (*M* = 1981 ms, *SD* = 593) than in both the survival scenario (*M* = 2472 ms, *SD* = 859), *t*(117) = 2.89, *p* = .0045, *d* = 0.647, and the pleasantness condition (*M* = 2351 ms, *SD* = 800), *t*(117) = 2.18, *p* = .0314, *d* = 0.487, while these latter two conditions did not differ significantly from one another, *t*(117) = 0.71, *p* = .4761, *d* = 0.16. In addition, when encoding times were controlled for, there was a main effect of both encoding condition, *F*(2, 116) = 9.32, *p* = .0002, ${n}_{p}^{2}$ = .138 and encoding time, *F*(1, 116) = 4.4, *p* = .0381, ${n}_{p}^{2}$ = .037, with the survival scenario still exhibiting a memory advantage over the moving scenario, *t*(116) = 2.55, *p* = .0119, *d* = 0.591, and the pleasantness encoding condition, *t*(116) = 4.29, *p* < .0001, *d* = 0.962. There was also a nonsignificant difference between the moving and pleasantness conditions, *t*(116) = 1.62, *p* = .1073, *d* = 0.37.

#### Relevance ratings

There were significant differences in the relevance rating scores between the three encoding conditions, *F*(2, 117) = 38.38, *p* < .0001, ${n}_{p}^{2}$ = .396. Relevance scores were lower when words were rated in the moving scenario (*M* = 2.34, *SD* = 0.49) than either the survival condition (*M* = 3.07, *SD* = 0.42), *t*(117) = −7.82, *p* < .0001, *d* = −1.75, or the pleasantness condition (*M* = 3.02, *SD* = 0.34), *t*(117) = −7.34, *p* < .0001, *d* = −1.64. However, the difference between the survival and pleasantness conditions was not reliable, *t*(78) = 0.48, *p* = .6324, *d* = 0.107. An analysis of covariance (ANCOVA), with the relevance rating scores as covariate, did not reveal a main effect of this factor on recall rates, *F*(1, 116) = 2.94, *p* = .0893, ${n}_{p}^{2}$ = .025. At the same time, the main effect of the encoding conditions was still significant, *F*(2, 116) = 9.43, *p* = .0002, ${n}_{p}^{2}$ = .14. However, while the mnemonic advantage of the survival scenario over the pleasantness encoding condition continued to be significant, *t*(116) = 4.33, *p* < .0001, *d* = 0.97, the difference between the survival and the moving scenarios was now not significant, *t*(116) = 1.58, *p* = .117, *d* = 0.436, and the positive difference between the moving scenario and the pleasantness condition was close to significance, *t*(116) = 1.98, *p* = .0505, *d* = 0.534.

#### Feeling real ratings

There were significant differences in the feeling real scores between the three encoding conditions, *F*(2, 117) = 7.38, *p* = .001, ${n}_{p}^{2}$ = .112. The scores were higher in the pleasantness condition (*M* = 4.83, *SD* = 1.52) than in either the survival scenario (*M* = 4.13, *SD* = 1.24), *t*(116) = 2.05, *p* = .0428, *d* = 0.458, or the moving scenario (*M* = 3.51, *SD* = 1.78), *t*(116) = 3.84, *p* = .0002, *d* = 0.859, while the difference between these two latter conditions failed to reach significance, *t*(116) = 1.79, *p* = .0757, *d* = 0.4. An ANCOVA with “reality” scores introduced as a covariate factor revealed no main effect of this factor on recall rates, *F*(1, 116) = 2.45, *p* = .1201, ${n}_{p}^{2}$ = .021, while the main effect of the encoding conditions remained significant, *F*(2, 116) = 11.05, *p* < .0001, ${n}_{p}^{2}$ = .16, with the recall rate being higher in the survival scenario than in the other two conditions—that is, moving:* t*(116) = 2.88, *p* = .0047, *d* = 0.653; pleasantness: *t*(116) = 4.61, *p* < .0001, *d* = 1.05. The difference between the moving and pleasantness conditions was not significant, *t*(116) = 1.68, *p* = .0963, *d* = 0.398.

### Discussion of Study 1

Study 1 replicated the survival processing advantage by presenting words in a visual display, with the aim of creating a greater sense of presence than in the traditional encoding situation where words are presented at the center of the screen without any visual display (Bonin et al., 2024; Nairne et al., 2007, 2008). Moreover, as in Bonin et al.’s (2025a) study, we used standardized material that was moderately relevant to survival issues, such as protecting from predators, avoiding pathogens, and finding steady supplies of food and water. In a previous study, we observed that words highly relevant to survival problems were recalled better than words of low relevance, and this was the case in both the survival and pleasantness encoding conditions (Bonin et al., 2024). Here, as in Bonin et al.’s (2025a) study, a survival processing advantage was observed in recall rates for moderately survival-relevant words. Further analyses revealed that the survival processing advantage on recall rates was not dependent on reaction times, despite the time taken to rate the words having an effect on recall rates. Regarding relevance ratings, we observed that the moving condition resulted in lower scores than the survival condition. When these scores were taken into account in a covariance analysis of recall rates, the survival advantage turned out to be nonsignificant compared with the moving condition, although it remained significant compared with the pleasantness condition. Could the survival effect in memory in this instance be due to a “congruity” effect? We will return to this point in the Discussion of Study 2.

An analysis of clustering (as indexed by ARC scores) revealed no reliable differences between the three encoding conditions, suggesting that none of them generated more relational processing (see Bonin et al., 2025a; Nairne & Pandeirada, 2008, for studies that also failed to find reliable differences in ARC scores between survival and pleasantness encoding conditions). Also, there were no more extralist intrusions in the survival condition than in the moving and pleasantness conditions (for similar findings, see Forester et al., 2020; Otgaar et al., 2015). Finally, the “feeling real” ratings differed significantly between the pleasantness condition and the other two scenarios, but failed to predict recall rates.

## Study 2: Survival effect in an experimental VR context

Study 1 was a necessary preliminary step in addressing the question of what happens when participants are immersed in a VR encoding situation. Although the survival effect has already been investigated in VR by Wang et al. (2023), this study is the only one of its sort and replication was therefore warranted given the replicability crisis in psychology (Asendorpf et al., 2013; Pashler & Harris, 2012). In the following study, we used one of the latest virtual reality technologies, a Meta Quest 2 headset running Unity3d software. This offers a higher level of detail than the DK2 headset used by Wang et al. (2023). This augmented realism should reinforce the survival effect in memory or, by contrast, provide a boost in both types of encoding context compared with the more conventional screen-based procedure. Importantly, Wang et al. (2023) did not compare recall rates obtained using virtual reality with those obtained using a traditional desktop setup.

### Method

#### Participants

A sample of 84 healthy participants (74 women, *M* = 16.65 years, *SD* = 1.62) with normal vision from Université Bourgogne Europe in Dijon took part in the study. (Four further participants were excluded from the analyses because their reaction times were significantly longer than those of the other participants.) The participants were randomly assigned to one of the two groups (*n* = 42 in each group): survival versus moving. The number of participants was chosen on the basis of Wang et al.’s (2023) Experiment 1A. Given a Cohen’s *d* value of 0.71 computed from Wang et al.’s (2023) Table 1 (p. 134), G*Power (Version 3.1.9.7; Faul et al., 2007) revealed that a sample of 66 participants (33 per group) was necessary in order to obtain a power of .8 (two-tailed; *α* = .05) in an independent *t* test. With such an effect size and threshold, 42 participants per group guaranteed an even greater power of .9. All participants were native speakers of French and none were taking medication known to affect the central nervous system. Participants received course credits for their participation. All the study procedures were approved by the Statutory Ethics Committee of the University Bourgogne Franche-Comté.

#### Apparatus

The experiment was performed using a virtual reality system equipped with a Meta Quest 2 headset (Meta, Menlo Park, CA). The Meta Quest 2 has a higher resolution than the DK2 headset used by Wang et al. (2023; i.e., 1832 × 1,920 pixels per eye vs. 960 × 1,080 pixels), and also has higher refresh rate (up to 120 Hz vs. 75 Hz for the DK2). The field of view was approximatively 90° and the latency was between 20 and 30 ms. These improvements guarantee smoother, more detailed images, thereby reducing visual fatigue and the screen door effect often observed with the DK2 headset. In terms of audio capability, the Meta Quest 2 features integrated speakers offering 3D spatial sound without the need for an external audio system, unlike the DK2 which needed external speakers. Unity3d (Unity Technologies, Inc., Shanghai, v2021.3.28f1) software was used to create the 3D environments. We used Unity’s maximum rendering mode, HDRP (High Definition Render Pipeline). The headset was connected to a powerful computer (Intel Core i7-14700 [28 CPUs], ~ 2.1GHz, RAM 32.0 GB) with a graphics card (NVIDIA GeForce RTX3080) to calculate the image, as the headset alone does not have enough power to display complex environments.

#### Materials

**Fig. 2:**
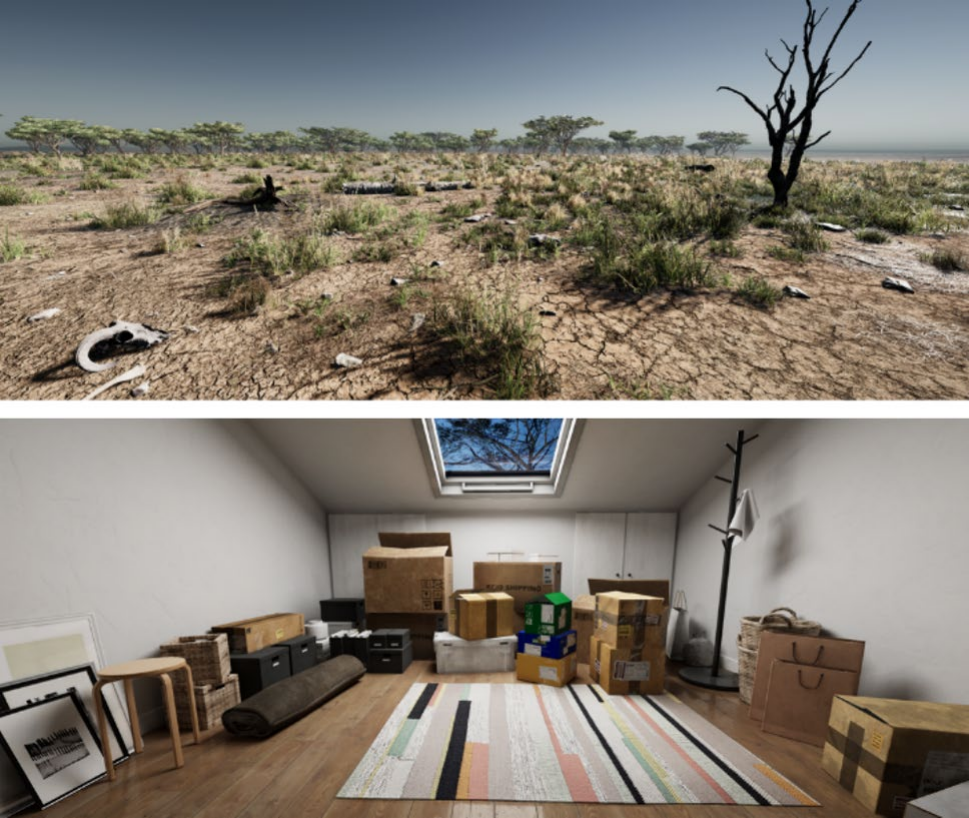
Illustration of the two encoding contexts used in Study 2: the survival (African savannah) context at the top and moving context at the bottom

Four virtual reality environments were created. Two of these were training/exposure environments, which were experienced by all participants. One depicted a living room in a domestic setting, and the other depicted a sunlit, snowy forest in winter (see Supplementary Material). The other two environments were used in the main phase of the experiment, and each participant only saw one of them depending on condition. The African savannah environment was used for the survival scenario, while the home environment was used for the moving condition (see Fig. 2). The ancestral environment displayed an African savannah on a hot day. The scene was sunny and cloudless. The cracked, drought-stricken ground was composed of dry grass, stones, bones, dead wood and acacia trees stretching to the horizon. Sounds such as wind and insects (e.g., cicadas, crickets) were added to encourage immersion (see Fig. 2). The home environment for the moving encoding condition showed a room with a closed door and boxes and other items, such as frames, rugs and bags on the floor. The room was well lit, with light coming through the skylight. Outdoor traffic sounds were added to reinforce the sense of an urban environment (see Fig. 2).

#### Procedure

The experiment was divided into two phases. The first phase comprised a training task and a word rating task. The second phase, which occurred immediately afterwards, comprised a rating task, interference tasks, surprise free recall, and completion of the physical presence subscale of the Multimodal Presence Scale (Makransky et al., 2017).

When they arrived at the laboratory, the participants were asked to complete a consent form and answer some demographic questions: age, level of education, native language, current use of neuroleptic medication, and familiarity with virtual reality devices. Next, the experimenter helped the participants put on the virtual reality headset. They could choose which controller to use to rate the words (left or right) depending on their dominant hand. To familiarize participants with the setup and limit their surprise at experiencing virtual reality (mainly present among novice users; Makransky et al., 2017), two training/exposure environments were randomly presented for 1-min each (i.e., snowy forest, living room, see Fig. A1 in the Supplemental Material A), and the participants were told to observe the surroundings. During this phase, participants had the opportunity to turn 360° to fully explore the scene and take one step to each side. A neutral 3D environment was then presented together with a 5-point Likert scale (see Fig. A2 in the Supplemental Material A). Participants were instructed to point to the different numbers and press the trigger on the controller to confirm their choice. To accustom them to the main rating phase, a word not present in the encoding list was presented at the bottom of the scale. They had to point to and confirm all the ratings three times in total.

During the subsequent encoding phase, the participants were randomly assigned to one of the two conditions: the African savannah or the home environment. The corresponding instructions were presented both visually and auditorily via the headset. These were very similar to the instructions in Study 1. The only exception related to the rating task, which was performed using 5-point Likert scales controlled in exactly the same way as in the training phase (see Fig. A2 in the Supplemental Material A). Participants were also informed that the words and scale would appear when they heard a certain sound. The participants were then immersed in their environment for 1 min. When the sound signal was heard, words were randomly presented at the center of their visual field, even if they moved their heads. They were then instructed to perform the word relevance task. The device was then removed, and the remainder of the procedure followed the same pattern as Study 1. The participants performed two interfering tasks for 3 min, after which they had 5 min to write down the previously presented words in any order they liked. To measure their sense of presence in the VR environment, the participants completed the physical presence subscale of the Multimodal Presence Scale (MSE; Makransky et al., 2017). The MSE consists of 15 questions subdivided into three subscales: physical presence, social presence, and self-presence. For the purposes of this study, only the five questions relating to physical presence were used (e.g., “The virtual environment seemed real to me”; “While I was in the virtual environment, I had a sense of “being there”). Participants were asked to respond to the statements using a 5-point Likert scale (1 = “strongly disagree”; 5 = “totally agree”). Finally, the participants were debriefed about the research objectives. The entire experimental session lasted approximately 30 min.

### Results

All means (and standard deviations) for correct recall percentages, encoding times, relevance ratings, and physical presence scores are presented in Table 1.

**Table 1:** Correct proportions of recall, encoding times (ms), relevance ratings and physical presence (both from 1 to 5) in Study 2 (means and standard deviation in brackets)

	Survival (African savannah)	Moving
Correct recall rates	.56 (0.12)	.43 (0.09)
Encoding times	2,756 (662)	2,474 (655)
Relevance ratings	2.86 (0.38)	2.30 (0.55)
Presence ratings	3.62 (0.58)	3.75 (0.48)

#### Correct recall and intrusions

A significant mnemonic advantage was found when words were rated in the survival scenario rather than in the moving scenario, *t*(82) = 5.41, *p* < .0001, *d* = 1.18. There were few extralist intrusions (*M* = .95, *SD* = 1.23), with similar proportions in both conditions (survival: *M* = 0.93, *SD* = 1.30; moving: *M* = 0.98, *SD* = 1.18), *t*(82) = 0.18, *p* = .8606, *d* = 0.038.

#### Clustering

As in Study 1, the ARC scores were above zero in both conditions—survival: *t*(41) = 5.22, *p* < .0001, *d* = 0.805, *M* = .21, *SD* = 0.27; moving: *t*(41) = 3.04, *p* = .0041, *d* = 0.47, *M* = .12, *SD* = 0.25—and there were no reliable differences between them, *t*(82) = 1.72, *p* = .09, *d* = 0.37. Including the semantic category classification of intrusions in the calculation of the ARC scores did not alter the pattern of results within or between conditions.

#### Encoding times

There was no significant difference in the time taken to rate the words between the encoding conditions, *t*(82) = 1.97, *p* = .0526, *d* = 0.43.

#### Ratings

Ratings were higher in the survival than in the moving condition, *t*(82) = 5.41, *p* < .0001, *d* = 1.18. However, an ANCOVA with rating scores as the covariate did not reveal a main effect of rating scores on recall rates, *F*(1, 81) = 0.01, *p* = .9373, ${n}_{p}^{2}$ = .00, with the mnemonic advantage of the survival scenario being significant, *F*(1, 81) = 21.67, *p* < .0001, ${n}_{p}^{2}$ = .211. It is interesting to note that exactly the same pattern of results was found when controlling for encoding times alone or in addition to the ratings.

#### Multimodal Presence Scale

The physical presence scores did not differ significantly between the two encoding conditions, *t*(82) = −1.13, *p* = .2601, *d* = 0.247.

#### Combined analyses: Virtual reality and classic encoding conditions

The recall rates, encoding times, relevance ratings, and physical presence scores were considered as dependent variables in the ANOVAs, which included type of setting (classic vs. virtual reality) and encoding condition (survival vs. moving) as between-subjects independent variables.

#### Correct recall, intrusions, and clustering

**Fig. 3:**
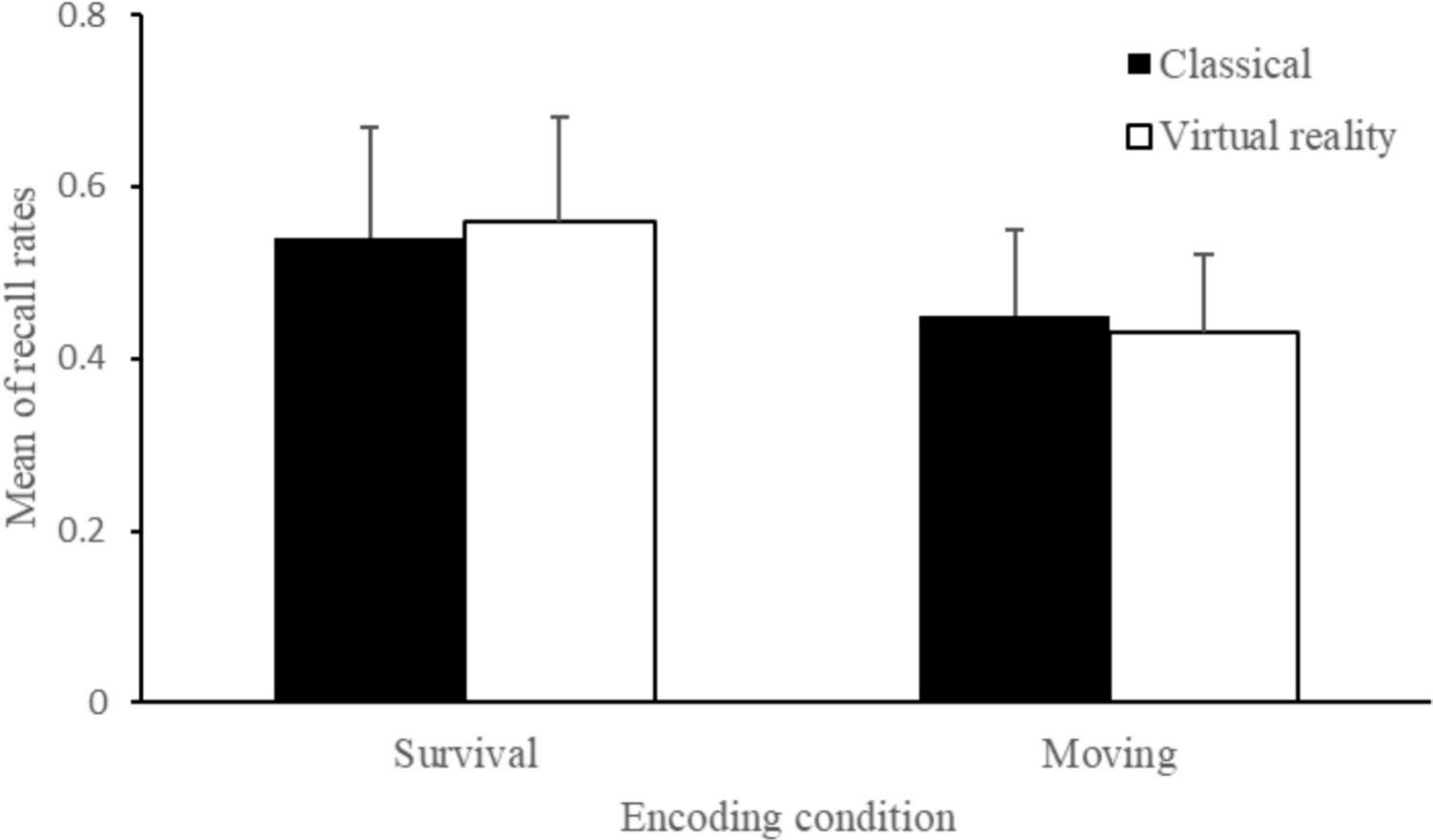
Recall rates as a function of type of experiment (classical vs. virtual reality) and encoding condition (survival vs. moving)

Recall rates were higher in the survival condition than in the moving condition, *F*(1, 160) = 37.59, *p* < .0001, ${n}_{p}^{2}$ = .190 (see Fig. 3). The type of setting, *F*(1, 160) = 0.05, *p* = .8168, ${n}_{p}^{2}$ < .001, and the interaction effect, *F*(1, 160) = 1.13, *p* = .2895, ${n}_{p}^{2}$ = .007, were not significant (Fig. 3). Given the null effects of type of setting and the interaction between type of setting and encoding condition, we ran Bayesian analyses with JASP (Version 0.95.3; JASP Team, 2025). These analyses indicated that the best model was the one that included only the encoding conditions (*BF*_*10*_ > 10^6^ with a null model including no effects), for which the data were roughly six and sixteen times more likely than in the models that included (1) the effect of type of setting, (2) all effects (*BF01* = 5.9 and *BF*_*01*_ = 16.42 with a null model including only the conditions effect).

There were no more intrusions in the survival condition than in the moving condition, *F*(1, 160) = 0.22, *p* = .6433, ${n}_{p}^{2}$ = .001. The type of setting, *F*(1, 160) = 1.33 , *p* = .2498, ${n}_{p}^{2}$ = .008, and the interaction effect, *F*(1, 160) = 0.04, *p* = .8355, ${n}_{p}^{2}$ < .001, were not significant.

ARC scores were not higher in the survival condition than in the moving condition, *F*(1, 160) = 3.06, *p* = .082, ${n}_{p}^{2}$ = .019. The type of setting, *F*(1, 160) = 0.13 , *p* = .7173, ${n}_{p}^{2}$ = .001, and the interaction effect, *F*(1, 160) = 0.27, *p* = .6008, ${n}_{p}^{2}$ = .002, were not significant.

#### Encoding times and ratings

Encoding times were longer in the survival condition than in the moving condition, *F*(1, 160) = 12.58, *p* = .0005, ${n}_{p}^{2}$ = .073. The effect of type of setting was also significant, *F*(1, 160) = 12.67, *p* = .0005, ${n}_{p}^{2}$ = .073, with it taking longer to rate the words in a virtual environment than in a classic desktop display. The interaction effect was not significant, *F*(1, 160) = 0.92, *p* = .3396, ${n}_{p}^{2}$ = .006. The ratings were higher in the survival condition than in the moving condition, *F*(1, 160) = 79.31, *p* < .0001, ${n}_{p}^{2}$ = .331. The type of setting, *F*(1, 160) = 2.89, *p* = .0909, ${n}_{p}^{2}$ = .018, and the interaction effect, *F*(1, 160) = 1.51, *p* = .2211, ${n}_{p}^{2}$ = .009, were not significant.

#### Physical presence

To compare the presence scores in the two studies, the values initially obtained with a 7-point scale in Study 1 were adjusted to a 5-point scale. It should be noted, however, that this comparison is essentially descriptive, as presence was measured using a single question in Study 1 (and was defined as “feeling real”), whereas physical presence was indicated by a composite score in Study 2. The means of the posttest questionnaire were not higher in the survival condition than in the moving condition, *F*(1, 160) = 0.99, *p* = .3219, ${n}_{p}^{2}$ = .006. The type of setting, *F*(1, 160) = 38.73, *p* < .0001, ${n}_{p}^{2}$ = .195, was significant, with participants expressing more physical presence in a virtual environment than in the traditional setting. The interaction effect was also significant, *F*(1, 160) = 4.1, *p* = .025, ${n}_{p}^{2}$ = .025, with the difference between the two settings being greater in the moving than in survival condition. An ANCOVA with physical presence as a covariate did not reveal a main effect^3^ of physical presence on recall rates, *F*(1, 159) = 0.01, *p* = .92, ${n}_{p}^{2}$ = .001, whereas the mnemonic advantage for the survival condition was still significant, *F*(1, 159) = 37.11, *p* < .0001, ${n}_{p}^{2}$ = .189, and no other effects reached significance.

### Discussion of Study 2

In Study 2, the survival processing advantage was observed in VR environments. As in Wang et al. (2023), words processed in a survival-related virtual environment were recalled better than when the same words were processed in a moving encoding situation. One novel aspect of our research was that it compared the survival effect observed with VR technology and a desktop display. The survival processing advantage in VR was no greater than that observed with a more conventional demonstration of this effect. Immersion therefore appears to be irrelevant to the survival processing advantage. Furthermore, it should be emphasized that similar encoding times for rating the words, number of intrusions, clustering scores, and physical presence as indexed by the MPS (Makransky et al., 2017) were found in the two conditions.

As in Study 1, relevance ratings were higher when words were processed in a survival scenario than in a moving scenario. This result has already been observed in the literature on the survival effect in memory (e.g., Dewhurst et al., 2023; Kroneisen et al., 2024; Nairne & Pandeirada, 2010; Nairne et al., 2007; Röer et al., 2013; Saraiva et al., 2021), and it has even been qualified as typical/common (Kroneisen & Bell, 2018; Röer et al., 2013). While the covariance analysis in Study 1, which took account of relevance ratings, showed that the survival effect in free recall was not significant when compared with the moving condition, this was not the case in Study 2. Given that Study 1 suggested that relevance ratings could mediate the survival effect, we conducted supplementary analyses in which we included these ratings as a mediator in a mediation model, using the conditions as a predictor and recall rates as the dependent variable. We used the PROCESS SPSS macro (Hayes, 2022) to run the analyses with 10,000 bootstrapped samples and 95% confidence intervals (CIs). The indirect effects of the conditions failed to reach significance when comparing the survival and moving conditions, *b* = 0.034, CI [−0.006, 0.081], and also the survival and pleasantness conditions, *b* = 0.11, CI [−0.09, 0.011]. The direct effects tests were the same as those used for the covariance analysis. Additionally, given that the pleasantness condition was somewhat different from these two conditions in that it was more similar to an “emotional” evaluation, we also ran an analysis restricted to the survival and moving scenarios and found that the indirect effect of the conditions was once again not significant, *b* = .023, CI [−.022, .073], while the direct effect just failed to reach significance, *b* = .064, *p* = .0564. In summary, the results of these supplementary analyses do not allow us to conclusively establish that relevance ratings mediate the survival effect observed in Study 1. The inconsistent relationship between relevance ratings and recall scores observed here for the survival and moving conditions has already been reported in the literature (e.g., Wang et al., 2023). More generally, these results are consistent with the debate in the literature concerning the role of congruence/relevance, which has led researchers to conclude that, while this factor may influence memory performance, it cannot fully account for the survival effect in memory (Erdfelder & Kroneisen, 2014; Nairne & Pandeirada, 2010).

## General discussion

As Thomas and Raymond (2022) write: “The living conditions of our ancestors were very different from our own, with habitats that were generally more dangerous. Predators, venomous animals of all kinds, poisonous plants and rival gangs were omnipresent threats in their daily lives. Added to these risks were the numerous possibilities of food or water shortages, or the risk of contracting an infectious disease” (p. 181, our translation). According to evolutionary psychologists, the selective pressures encountered by our hunter–gatherer ancestors, such as the need to find food and water, protect themselves from predators and avoid pathogens, have left their mark on our cognition. In order to cope with these pressures, natural selection crafted a powerful memory system to help us remember where to find food and drinking water and where to avoid dangerous animals (Nairne, 2016, 2022). Indeed, in the distant past, failure to remember where to find food or drinking water could lead to death, as could forgetting where dangerous or venomous animals might lurk. But how can we reveal the cognitive, emotional and motivational mechanisms forged by these selective pressures? When it comes to the functional characteristics of our memory, which were shaped by the selective pressures our hunter–gatherer ancestors experienced, researchers have developed some ingenious experimental techniques. The survival effect is a case in point.

As described in the Introduction, the survival effect in memory refers to the fact that words (as well as pictures, Dewhurst et al., 2023; Otgaar et al., 2010; and faces, Hou & Liu, 2019) that are processed in relation to survival are remembered better than other items. The survival effect has been found in laboratory experiments involving desktop displays. In experiments designed to demonstrate the survival effect in memory, participants are typically instructed to imagine themselves in a survival situation (ancestral or modern). They are then presented with words on a standard computer screen and asked to rate their relevance to the imagined scenario. After a period of time—usually just a few minutes (though see Raymaekers et al., 2014, for evidence of a survival effect after several days)—they are asked to recall the words.^4^ Needless to say, there is a significant difference between the situation of our ancestors, who relied on their memory to locate food and water, and avoid potential dangers, and the way in which this same memory is tested in “artificial” situations among people living in completely different environments. This is why the ecological validity of the results obtained is a recurrent question in the field of adaptive memory. Wang et al. (2023), who are the only researchers to date to have designed a virtual reality study of the survival effect, have claimed that “the survival VREs [Virtual Reality Environments] offer researchers the opportunity to design a survival processing study with good internal validity and qualified ecological validity” (p. 130). Almost all of the results obtained on the survival processing advantage are based on laboratory techniques, and are therefore “artificial” in nature. This is why virtual reality techniques offer new ways to investigate memory in general and adaptive memory in particular.

Given that VR can enrich the sensory quality of the tested environments and the intensity of the lived experience, it is reasonable to think that it can recreate some of the conditions in which our ancestors lived, or, more generally, survival contexts, whether modern or ancient, better that the participants’ imagination alone. While Wang et al. (2023) demonstrated that the survival effect could be obtained in savannah and battlefield simulated environments, we aimed to go a step further in our study. Firstly, we aimed to replicate this effect in a virtual reality setting, using more advanced technology that enhances immersion. Secondly, and this constitutes one of the original features of our research, we compared the VR and classic computer screen presentations using the same stimuli. What do the findings tell us?

Perhaps counterintuitively, when we compare the two situations—virtual reality and the classic computer screen—the survival effect (as measured by correct free recall rates) was no greater in the former than the latter. We predicted that placing individuals in a richer, more stimulating environment would lead to more elaborate processing and therefore the creation of richer memory traces. This would result in the retrieval of a greater number of words than in the classic desktop situation. As mentioned in the Introduction, a considerable amount of work has been conducted in this area. It has been demonstrated that the survival scenario permits the generation of more ideas than the moving scenario (Röer et al., 2013). Ideas generated in the former scenario are also rated as being more creative than those generated in the latter (Bell et al., 2015). However, the results of the present study suggest that the “context” in which survival encoding takes place is not of fundamental importance for obtaining a survival effect. These results are consistent with a meta-analysis by Tay et al. (2019), which showed that grassland contexts per se may have a weaker effect than the presence of a survival threat. In other words, the important thing is that the words are processed in relation to a survival issue. The proximate mechanisms are triggered by cues evocative of survival or an explicit request for survival processing (Klein, 2013) and exert their effects. Therefore, an a priori better context, such as a virtual environment in which a savannah landscape can be viewed at 380°, with visual and sound elements evocative of the difficulty of finding food and drinking water and of the possibility of dangerous animals being present (i.e., an environment capable of generating a convincing ancestral context) does not seem to lead to the optimal mobilization of the mechanisms responsible for the survival effect any more than an environment essentially resulting from the participants’ imagination.

Is it possible that the use of images in Study 1 reduced the “spontaneous” use of mental imagery in the standard situation, in which there was no visual support and only text was used, and that we therefore ultimately chose an encoding condition that was less likely to reflect a difference in survival effect compared with the VR context?^5^ Indeed, in the standard situation for demonstrating the survival effect, there is no visual support and the scenarios are therefore based solely on textual information, consequently requiring an open-ended interpretation of the survival (and moving) scenarios. The data on the impact of imagery on the survival effect, are mixed: Nouchi’s (2011) results showed a greater survival effect in individuals with high than with low mental imagery ability, while the findings of Kroneisen et al. (2013) fail to show that interactive imagery (i.e., imagining using objects in the grasslands scenario vs. in the moving scenario) plays a role in this effect. Fortunately, data are available to contribute to the discussion on this issue. In a recent study, Bonin et al. (2025a) compared the survival effect measured on the basis of commonly used graded word relevance judgments (i.e., using Likert scales) and of binary decisions (i.e., is the word relevant in this situation (yes/no)? The words used in this comparative study were exactly the same as those used here. In Bonin et al.’s Study 1, the survival effect was obtained with ratings and, importantly, was measured in response to the *standard “text” presentation* of the survival scenario. We wanted to compare this situation with those used in the present study. We therefore conducted an item-by-item analysis in which we were able to compare the survival effect in the “text” condition (Study 1 by Bonin et al., 2025a) with that obtained in the ‘image’ condition (Study 1) and “virtual reality” condition (Study 2). Detailed analyses are provided in the Supplemental Material B. Several interesting results emerged. Firstly, the type of experiment (i.e., the setting) had no impact on either the survival encoding or the control conditions used in the experiments. Secondly, survival encoding led to higher recall, regardless of the control condition employed (pleasantness vs. moving). Thirdly, the effects were quite similar in the different experiments and, therefore, in the different types of setting (see Fig. B1 in the Supplemental Material B). We can therefore reject the idea that the decision to present images in addition to the words in Study 1 was possibly unsuitable as a way of comparing the survival effect in virtual reality, as well as the idea that a standard “text” presentation would have been more suitable.

The present results are therefore consistent with previous findings which have shown that ancestral environments are no more effective in boosting the survival effect than modern environments (e.g., Soderstrom & McCabe, 2011), or environments as diverse and unfamiliar as outer space (Kostic et al., 2012). The results of the Moura et al. (2021) study tells a similar story. To determine whether adaptive memory has ancestral priority, Moura et al. (2021) examined memory performance in adults in survival situations in the ancestral environments of savannah, rainforest and deciduous forest, as well as in the modern environments of coniferous forest, desert, tundra and an urban environment. Participants were asked to rate words for their relevance to the survival problem of finding and using medicinal plants to treat a disease, followed by a surprise word recall test. The results showed no ancestral priority in the recall of relevant information, as recall was similar in both the ancestral and modern environments.

Given the current state of knowledge, one could argue that the “ecological context”—a term covering the methodology used to study the survival effect—contributes little when it comes to obtaining a survival effect. This would explain why this effect can be obtained simply by asking participants to rate words to “try to stay alive” without providing any additional information about the context (Klein, 2013). It could be argued that these types of findings call into question the evolutionary interpretation of the survival effect, and more broadly, the adaptive memory view. In contrast, it could be said that most evolved mechanisms are, to some extent, “independent” of the contexts in which they emerged and that they are activated by specific cues. For example, avoidance reactions and/or disgust have been observed in response of individuals displaying signs of disease, such as pustules on the face (Bonin et al., 2025b). However, very similar reactions have also been observed in participants viewing individuals with wine stains on their face that resemble disease symptoms (e.g., Ryan et al., 2012). Such results, including the current ones, do not necessarily call into question the evolutionary account of survival effects.

There has probably not yet been enough work done in VR on the influence of immersion and presence on memory performance to allow a clear understanding of their respective effects. In line with Cadet and Chainay’s (2020) findings, our data does not suggest that a higher level of immersion (i.e., head-mounted display vs. computer screen) results in better memory performance (see also Makransky et al., 2019). The presence/feeling real ratings are also interesting. It is important to note that the studies available in the literature on the survival effect have so far failed to reveal any characteristics of the survival scenario that contribute, at least in part, to this effect. For instance, arousal (Kang et al., 2008), valence (Yang et al., 2014), mortality salience (Bell et al., 2013; Bugaiska et al., 2014) triggered by the survival scenario do not appear to play a role in the survival effect (Erdfelder & Kroneisen, 2014, for a review). The level of perceived threat, however, plays a role in survival effect (Olds et al., 2014).^6^

Other researchers had already questioned the ecological validity of the survival effect in memory before any virtual reality studies on this topic were carried out. We believe that virtual reality is not the only way to increase the ecological validity of research into adaptive memory. As Schwartz (2019) points out, the ecological validity of the survival effect can be questioned given that humans in the distant past clearly did not explicitly assess objects for their relevance to their own survival. As Schwartz et al. (2024) suggest, a better understanding of the survival effect may require studies of nonhuman primates in the wild, although such studies will necessitate the use of ingenious methodologies. In particular, comparative studies of the survival processing advantage between human and nonhuman primates should help us to a better understanding of the evolutionary roots of this effect (see Schwartz et al., 2024, for avenues of research).^7^ In the same vein, another way to study the survival processing advantage might be to compare individuals from modern civilizations with those still living as hunter–gatherers. To illustrate, Barrett and Broesch (2012) investigated Shuar children from the Amazon region of Ecuador and city-dwelling children from Los Angeles. The children were asked to look at cards representing animals together with information about them (i.e., name, whether they are dangerous, what they eat). During the recall phase, the children were asked to provide information about each animal (e.g., whether it was dangerous). The researchers found that children in both populations remembered the dangerousness of the animals better than their diet or name, and that the U.S. American children’s danger learning effects nearly approximated those of the Shuar.

It is important to acknowledge some limitations of the present work in order to help and guide future work. First, the feeling of “presence” can undoubtedly be increased in future studies of the survival effect in virtual reality. In particular, the participants in our study were forced to remain static; however, in situations where individuals need to find food and drinking water and protect themselves from predators, they must move around in order to achieve precisely these goals. Future studies on the survival effect would benefit from allowing participants to move around and be physically active. This is particularly important given that physical activity was essential to our ancestors’ existence (Carrera-Bastos et al., 2025). In addition, a study by Sauzéon et al. (2012) showed that active exploration in a virtual environment significantly improves memory encoding and retrieval (see also Plancher et al., 2013). More precisely, Sauzéon et al. compared two modes of exploration in a virtual apartment containing objects to be memorized: active navigation (i.e., movement using a joystick or by walking) and passive navigation (i.e., a guided path in the form of a prerecorded video). Secondly, we only considered one type of simulated survival environment, but it would be interesting to examine others, such as rainforests, deciduous forests, or even more modern, fantasy, or futuristic environments. It would also be interesting to focus on one simulated environment at a time (e.g., surviving vs. moving) and to vary the types of problems to be solved (e.g., finding food, protecting oneself from attackers). This is because some researchers have claimed that when survival scenarios have been restricted to a single type of survival problem, the survival processing advantage is no longer observed (Kroneisen & Erdfelder, 2011; but see Bonin et al., 2024). Thirdly, future studies may benefit from creating environments that elicit a greater sensation of presence through the use of situations that provoke stronger emotions, particularly fear. Gromer et al. (2019) found that the sensation of presence was greater in a highly realistic fear-inducing emotional environment (height exposure) than in a non-fear-inducing virtual environment (forest exposure), regardless of the level of realism (visual properties and sound presence). This suggests that the sensation of presence increases in a virtual environment in which animal cries or sudden tree creaks can be heard and where animals or characters may appear. Item memorization may therefore be greater in such an environment than in a less fear-inducing one.

In conclusion, we replicated the survival effect using virtual reality technology, something which had previously only been observed once in the literature (Wang et al., 2023). The finding that the survival processing advantage is no greater in a virtual reality setting than in a traditional desktop display situation suggests that this effect is more dependent on the activation of potential threats, such as a scarcity of food or drinking water, or the presence of predators, than on the “ecological” context in which these threats are implemented.

## Supplementary Information

Below is the link to the electronic supplementary material.

Supplementary file1 (PDF 1281 KB)

Supplementary file2 (PDF 164 KB)

## Data Availability

The datasets generated and/or analyzed during the current study are available from the corresponding author on reasonable request.

## Footnotes

1 We thank an anonymous reviewer for pointing this out to us.

2 The computations were done with G*Power (Version 3.1.9.7; Faul et al., 2007).

3 In addition, when included in the model, no interaction of the encoding condition and settings with physical presence was significant.

4 A few studies have used alternative experimental procedures such as remembering details of a character in a story involving survival or house-move (Cook et al., 2023), or recognizing sounds rated for their relevance in these two conditions (Watson, 2022).

5 We thank an anonymous reviewer for pointing this out to us.

6 As suggested by an anonymous reviewer, we cannot rule out the possibility that the moving condition in Study 2 was perceived as more “threatening” than the survival condition due to the presence of road traffic sounds (versus natural sounds in the survival condition). The results of the few existing studies on the impact of exposure to city-related sounds on memory are mixed, showing either a negative impact (e.g., Hygge et al., 2003) or no notable impact (e.g., Fejzic et al., 2025). In particular, Fejzic et al.’s (2025) study compared exposure to city-related or nature-related sounds and found no effect on item memory. If word memorization was negatively impacted by the city-related sound context of the moving condition in the present study, we would have expected to observe an increase in the survival effect in VR compared with the traditional desktop display. Also, a lower level of word memorization should have been obtained in the moving condition in VR than in the traditional condition, as the latter was not accompanied by sound. However, recall rates were almost identical in these two conditions, suggesting that the soundtrack used in the moving condition in VR had no significant effect on word memorization, although we cannot rule out the possibility that “traffic noise” was perceived as more threatening than the “nature-related” sounds presented in the survival context (e.g., wind).

7 For instance, research investigating avoidance behaviors toward sources of contamination in bonobos has shown that, like humans, they experience disgust, which enables them to avoid contaminated food (Sarabian et al., 2018).

## Declarations

### Author note

The authors would like to thank Eva Calandras and Gaston Vermorel for their help in collecting data for Studies 1 and 2.
