# Supplementary material for: The survival processing advantage in memory using virtual reality versus traditional desktop display: Does it make a difference?
Source: Mem Cognit. 2026 Feb 20;54(5):1900–16. doi: 10.3758/s13421-025-01846-2 (PMC13407459; doi:10.3758/s13421-025-01846-2)

Supplemental Material A. Illustration of the virtual environments used for training in Study 2 and the rating procedure used in the virtual environments

Training/exposure environments

Living room environment.. The living room scene showed a room in a normal house situation on a rainy day. Various pieces of furniture were present in the scene, including chairs, a sofa and storage cupboards. Decorative elements such as cushions, books and rugs were also added to fill the room. Finally, the sound of rain was included to make the indoor setting more appealing (see Figure A1).

Snowy forest. The snowy forest environment consisted of slender trees, rays of sunlight and large rocks. The ground was covered with snow and branches. The sound of birds and wind were added to make the setting more immersive (see Figure A1).

Figure A1

Illustration of the two training/exposure contexts used in Study 2 (snowy forest context at the top and living room context at the bottom)

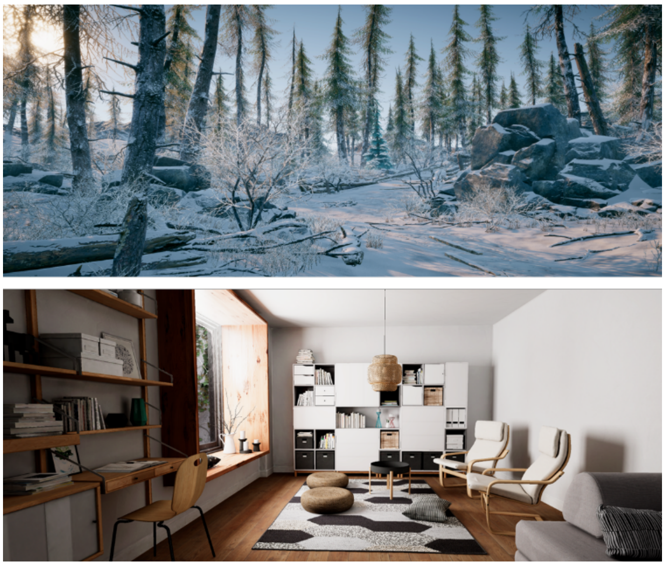

Figure A2

Illustration of the rating procedure using in the training phase. The French word "livre" means book

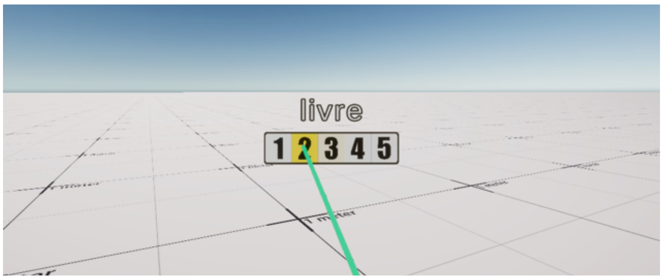

Supplement: Supplementary file 1 — Supplementary file1 (PDF 1281 KB) [file 13421_2025_1846_MOESM1_ESM.pdf]
