# Supplementary material for: The survival processing advantage in memory using virtual reality versus traditional desktop display: Does it make a difference?
Source: Mem Cognit. 2026 Feb 20;54(5):1900–16. doi: 10.3758/s13421-025-01846-2 (PMC13407459; doi:10.3758/s13421-025-01846-2)
Supplement: Supplementary file 2 — Supplementary file2 (PDF 164 KB) [file 13421_2025_1846_MOESM2_ESM.pdf]

Supplemental Material B. By-item analysis of correct recall rates obtained in (a) the "text" condition (Study 1 by Bonin et al., 2025a), (b) the 'image' condition (current Study 1) and (c) the "virtual reality" condition (current Study 2).

By-item paired *t*-tests were conducted on the recall rates obtained in the different settings and with the different encoding types in order to compare the type of experience (i.e., the setting) and the type of encoding factors, which are not completely crossed in the data from the three experiments.
There were no significant differences in the recall rates between the survival conditions, *Max(|t|)* = 1.13, *min(p)* = .2678, *Max(|d|)* = 0.16, or the control conditions, *Max(|t|)* = 1.74, *min(p)* = .0929, *Max(|d|)* = 0.34. By contrast, the survival effect was significant regardless of the setting or control condition used, *min(|t|)* = 3.01, *Max(p)* = .0054, *min(|d|)* = 0.55 (see Figure B1 for a representation of the survival processing advantage (SPA) for all possible comparisons).

After computing the by-item survival effect scores as the difference between the recall rates obtained in the survival condition in one setting and the recall rates in the control condition in the same or a different setting, additional paired *t*-tests were performed to compare the SPA when considering all possible combinations of settings and possible control conditions.

Regardless of which settings were compared, the SPA did not differ significantly when the moving condition was used as the control condition, *Max(|t|)* = 1.65, *min(p)* = .1093, *Max(|d|)* = 0.253. The same was true when the pleasantness condition was used as the control condition, *Max(|t|)* = 1.73, *min(p)* = .0947, *Max(|d|)* = 0.312. Additionally, only one significant difference was observed between the SPAs computed over two control conditions: that reported in Bonin et al.'s (2025a) Study 1 using the pleasantness control condition and the moving control scenario used in the current Study 1, $t(29)$ = 2.21, p = .0349, d = 0.521.

Figure B1

Survival Processing Advantage (SPA) computed by combining settings and control conditions

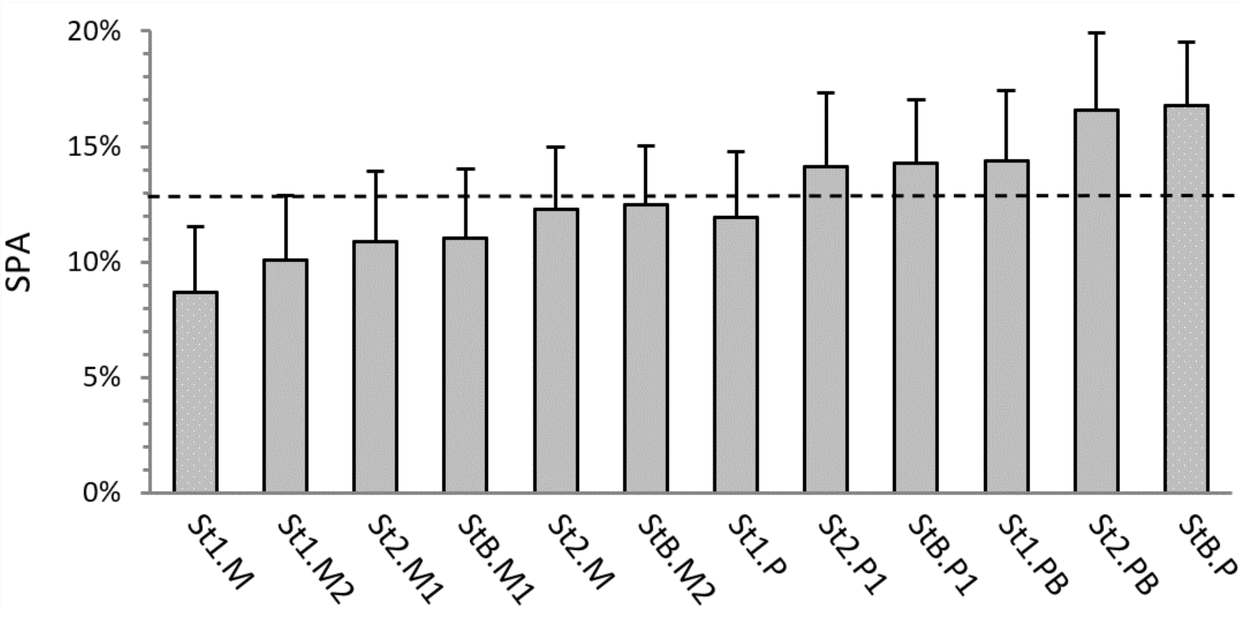

Notes. Each bar shows the difference in the means between the recall rate in the survival condition observed in one of the studies (St1 = Study 1, St2 = Study 2, StB = Study 1 by Bonin et al., 2025a) and the recall rate obtained in a control condition (M = moving, P = pleasantness), either in the same study or not (1, 2 and B are added after the control condition letter). The dashed line shows the mean across all SPAs, and the error bars correspond to the standard errors of the means.
